# Psychosocial Stress Induces Schizophrenia-Like Behavior in Mice With Reduced MMP-9 Activity

**DOI:** 10.3389/fnbeh.2019.00195

**Published:** 2019-08-29

**Authors:** Behnam Vafadari, Shiladitya Mitra, Marzena Stefaniuk, Leszek Kaczmarek

**Affiliations:** ^1^BRAINCITY, Nencki Institute, Warsaw, Poland; ^2^Chair and Institute of Environmental Medicine, UNIKA-T, Technical University of Munich, Helmholtz Zentrum München, Augsburg, Germany

**Keywords:** gene × environment (G×E) interaction, schizophrenia, negative symptoms, clozapine, antipsychotic, MMP-9

## Abstract

Understanding gene-environment interactions in the pathogenesis of schizophrenia remains a major research challenge. Matrix metalloproteinase-9 (MMP-9) has been previously implicated in the pathophysiology of schizophrenia. In the present study, adolescent *Mmp-9* heterozygous mice, with a genetically lower level of MMP-9, were subjected to resident-intruder psychosocial stress for 3 weeks and then examined in behavioral tests that evaluated cognitive deficits and positive- and negative-like symptoms of schizophrenia. Cognitive and positive symptoms in unstressed *Mmp-9* heterozygous mice were unaffected by stress exposure, whereas negative symptoms were manifested only after stress exposure. Interestingly, negative symptoms were ameliorated by treatment with the antipsychotic drug clozapine. We describe a novel gene × environment interaction mouse model of schizophrenia. Lower MMP-9 levels in the brain might be a risk factor for schizophrenia that, in combination with environmental factors (e.g., psychosocial stress), may evoke schizophrenia-like symptoms that are sensitive to antipsychotic treatment.

## Introduction

Schizophrenia is a neuropsychiatric disorder that affects approximately 0.5% of the human population (Saha et al., 2005). The disease is characterized by the heterogeneous display of positive symptoms (e.g., hallucinations, delusions, and thought disorder), negative symptoms (e.g., avolition, restricted affect, poverty of speech, and social withdrawal), and cognitive deficits (e.g., working memory deficits, executive dysfunction, and attentional impairments). The onset of schizophrenia typically occurs during early adulthood and is usually associated with lifelong disability (Lewis and Lieberman, 2000; Cirulli et al., 2009; Schmitt et al., 2014; Flores et al., 2016). Although multiple genes have been implicated in the etiology of schizophrenia (McGuffin et al., 2003; Hosak, 2013), no single genetic alteration has been able to satisfactorily explain all of its complex symptoms (Gottesman and Shields, 1967; Sanders et al., 2008). Thus, environmental factors have been suggested to interact with genetic liability to cause this disease (Tsuang, 2000; Mackay-Sim et al., 2003; McGrath et al., 2004; Owen et al., 2005; van Os et al., 2010; Hosák and Hosakova, 2015).

In stimulated neurons, matrix metalloproteinase-9 (MMP-9) is secreted from dendritic spines that harbor excitatory synapses (Wilczynski et al., 2008; Szepesi et al., 2014). MMP-9 has been shown to be involved in *N*-methyl-D-aspartate (NMDA) signaling and functional and structural synaptic plasticity (Huntley, 2012; Vafadari et al., 2016). These phenomena have been implicated in the pathophysiology of schizophrenia (Olney et al., 1999; Anand et al., 2000; du Bois and Huang, 2007; Penzes et al., 2011; Moyer et al., 2015). Recent studies of MMP-9 gene polymorphisms and MMP-9 mRNA and protein levels have implicated MMP-9 in schizophrenia patients (Rybakowski et al., 2009; Lepeta and Kaczmarek, 2015; Lepeta et al., 2017). *Mmp-9* knockout mice have also been shown to exhibit alterations of emotional and cognitive behaviors (Mizoguchi et al., 2010).

To investigate the possible role of environmental factors that act in concert with *Mmp-9*-dependent genetic predisposition in schizophrenia, we studied *Mmp-9* heterozygous knockout mice with low MMP-9 activity. The mice were subjected to psychosocial stress in a modified resident-intruder paradigm (Adamcio et al., 2009) and then tested in various behavioral paradigms to analyze cognitive deficits and positive- and negative-like symptoms of schizophrenia. Wild type (wt) mice were compared with *Mmp-9* heterozygotes, and stressed mice were compared with non-stressed mice. The stress procedure that was employed herein is considered to mimic at-risk disease conditions, such as social stress (e.g., urbanicity and social threat perception; Lewis et al., 1992; Kelly et al., 2010; Lederbogen et al., 2013; Haddad et al., 2015), bullying (Trotta et al., 2013), and adolescent trauma (Morgan et al., 2007).

## Materials and Methods

### Animals

**Figure 1:**
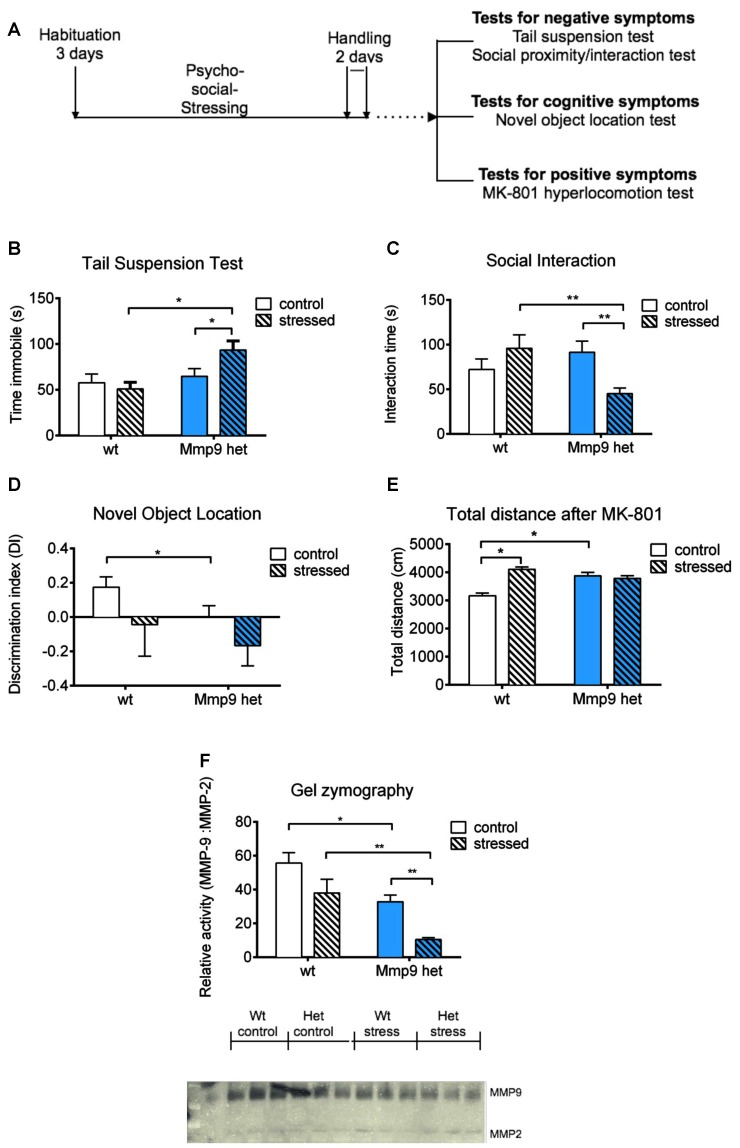
**(A)** Schematic diagram of the experiment. **(B)** Tail suspension test. Stressed *Mmp-9* heterozygous mice had a longer duration of immobility [individual *t*-test, *p* < 0.05; two-way analysis of variance (ANOVA), *F*_(1,37)_ = 7.008, *p* < 0.05] compared with their non-stressed counterparts and stressed wt mice. **(C)** Social interaction test. The time spent interacting with unfamiliar mice decreased in stressed heterozygous mice (individual *t*-test, *p* < 0.05; two-way ANOVA, *F*_(1,37)_ = 8.9555, *p* < 0.05) compared with their non-stressed counterparts and stressed wt mice. **(D)** Novel object recognition test. *Mmp-9* heterozygous mice spent less time with the novel object (individual *t*-test, *p* < 0.05) compared with wt mice. **(E)** Treatment with MK-801 increases the distance traveled in the open field in heterozygous mice and stressed wt mice (individual *t*-test, *p* < 0.05; two-way ANOVA, *F*_(1,37)_ = 14.61, *p* < 0.005) compared with non-stressed wt mice. **(F)** Gel zymography and relative densitometric plot of matrix metalloproteinase-9 (MMP-9) activity normalized to MMP-2 activity. MMP-9 activity significantly decreased (individual *U*-test, *p* < 0.05; two-way ANOVA, *F*_(1,8)_ = 13.9, *p* < 0.05) after stress exposure compared with the other groups. wt, wt mice; Het, *Mmp-9* heterozygous mice. *p < 0.05, **p < 0.01.

Mice were obtained from an in-house breeding facility at the Animal House at Nencki Institute. The experiments were conducted in 2-month-old, gender-matched *Mmp-9* heterozygous knockout mice on a C57Bl/6J background and wt C57Bl/6J littermates. For the induction of psychosocial stress, gender-matched adult FVB mice were used as the aggressor strain. Food and water were provided *ad libitum*. Temperature was maintained at 21–23°C with 50%–60% relative humidity and a 12/12 h light/dark cycle (lights on 7 AM–7 PM). All of the procedures were performed by experimenters who were blind to genotype and were in accordance with the Animal Protection Act in Poland (Directive 2010/63/EU) and approved by the 1st Local Ethical Committee on Animal Experimentation of the Faculty of Biology, Warsaw University (permission no. 148/2016 and 507/2018). The total number of mice that were used for the behavioral experiments after stress exposure was 41 (*n* = 8 control wt, *n* = 12 control *Mmp-9* heterozygotes, *n* = 9 stressed wt, *n* = 12 stressed *Mmp-9* heterozygotes). For the analysis of the effect of clozapine, the total number of mice was 22 (*n* = 5 control wt, *n* = 6 control *Mmp-9* heterozygotes, *n* = 5 stressed wt, *n* = 6 stressed *Mmp-9* heterozygotes). The vehicle control groups comprised 12 mice (*n* = 6 stressed wt, *n* = 6 stressed *Mmp-9* heterozygotes). Initially, the enzymatic activity of MMP-9 in brain extracts from heterozygous mice was assessed. The analysis revealed that MMP-9 levels in heterozygous mice were two-fold lower than in wt mice (Figure 1F).

### Psychosocial Stress

This procedure was adapted from a previous study (Adamcio et al., 2009). Individually housed wt mice and *Mmp-9* heterozygotes were introduced as intruders in the cage of gender-matched aggressor FVB mice. They were allowed to interact until the first attack by the aggressor FVB mouse. *Mmp-9* heterozygotes and wt mice were then placed under a perforated metal-cup cage to limit physical interaction for 1 h. To prevent habituation, a Latin-square distribution of the intruder and aggressor mice was used (Pryce and Fuchs, 2016). Briefly, experimental mice were introduced to different aggressors everyday that followed a circular combination pattern. The procedure lasted 21 days.

### Behavioral Tests

The behavioral tests were performed to assess cognitive deficits and positive and negative symptoms of schizophrenia. At the end of the behavioral tests, the mice were sacrificed for subsequent molecular analysis. In another series of experiments, after the mice were subjected to stress, they were treated with the atypical antipsychotic drug clozapine. One week after clozapine administration, the mice underwent behavioral testing. All movements were recorded with an overhead video camera and analyzed using Noldus Ethovision XT software for all of the behavioral tests, with the exception of the tail suspension test and social interaction test, which were video recorded for later analysis.

#### Analysis of Positive-Like Symptoms

MK-801 (dizocilpine maleate) is an NMDA receptor antagonist that induces pro-hallucinogenic effects in humans and hyperlocomotion in rodents (van den Buuse, 2010). MK-801-induced hyperlocomotion is employed to model and analyze schizophrenia-like behavior in rodents (Young et al., 2010; Jones et al., 2011). *Mmp-9* heterozygous and wt mice were placed in an open field apparatus (40 cm × 40 cm) and allowed free exploration for the first 30 min. The mice were then given an intraperitoneal injection of physiological saline and returned to the open field for 1 h. Afterward, the mice received MK-801 (0.25 mg/kg, Sigma) and were reintroduced to the open field and allowed free exploration for 1 h. All movements were video recorded and post analyzed with Ethovision XT software.

#### Analysis of Cognitive Deficits

The novel object recognition test is used to assess cognitive abilities in mice. The protocol that was used in the present study was adapted from a previous study (Vogel-Ciernia and Wood, 2014). The novel object recognition test and its variations have been used to assess cognitive symptoms in rodent models of schizophrenia (Jones et al., 2011; Stuchlik and Sumiyoshi, 2014; Grayson et al., 2015). The mice were placed in an open field apparatus with ~50 lux illumination and allowed free exploration for 10 min. Afterward, two similar objects were placed on the same axis near two corners of the apparatus. The mice were allowed to explore these two objects for 5 min and then they were returned to their home cage for 1 h. At the end of this period, one of the objects was moved to a new location in the apparatus (i.e., to the diagonal corner that was opposite to the other object). The mice were reintroduced to the apparatus and allowed to explore the objects for 5 min. All movements were video recorded and post analyzed with Ethovision XT software.

#### Analysis of Negative-Like Symptoms

The tail suspension test was designed to assess the effect of stress in mice, the results of which are considered to measure behavioral despair and the lack of motivation, characteristics of negative symptoms of schizophrenia (Porsolt et al., 1993). The mice were suspended by their tail for 5 min from the edge of a box with a white background. The distance between the mouse’s nose and the ground was 20 cm.

The social interaction test was developed to analyze anxiety-like behavior and traits of social avoidance in mice (Defensor et al., 2011). Social withdrawal is also a characteristic negative symptom of schizophrenia (Porsolt et al., 1993). Experimental mice and gender-matched novel wt mice (2–3 months old) of the same strain (C57Bl/6J) were introduced to a testing cage. They were allowed to interact with each other for 10 min. The total duration of interaction (e.g., nose to nose, nose to head, nose to ano-genital region) was analyzed manually.

### Clozapine Treatment

**Figure 2:**
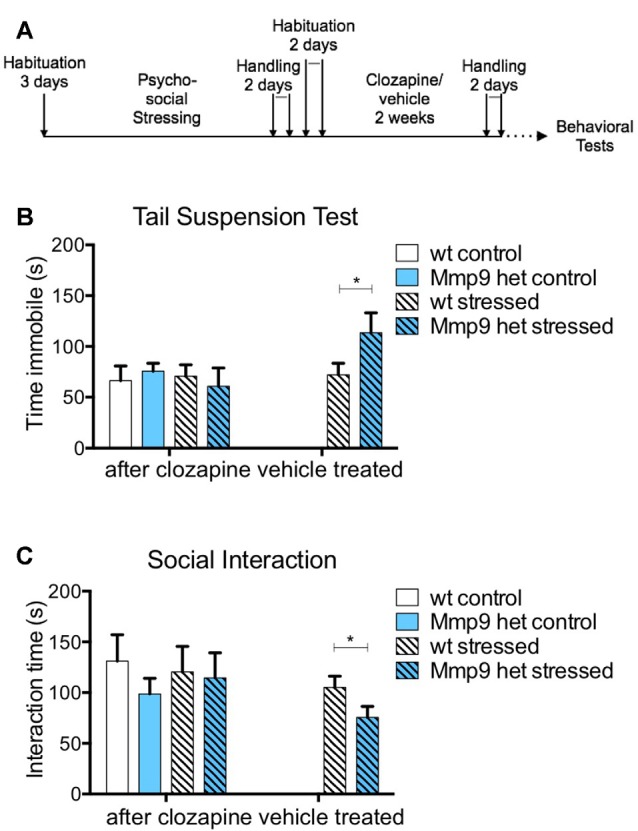
Effect of clozapine on negative symptoms. **(A)** Schematic diagram of clozapine treatment. **(B)** Clozapine treatment attenuated the increase in immobility in stressed *Mmp-9* heterozygous mice in the tail suspension test compared with their non-stressed counterparts and stressed wt mice. No effects of clozapine were observed in the vehicle-treated group (individual *t*-test, *p* < 0.05). **(C)** Clozapine ameliorated the differences in interaction time between stressed and non-stressed wt and heterozygous mice. Vehicle treatment alone did not exert any effects in stressed heterozygous mice, which exhibited a significant decrease in interaction time (individual *t*-test, *p* < 0.05) compared with wt mice. wt, wt control mice; Het, *Mmp-9* heterozygous mice. *p < 0.05.

Clozapine is an atypical antipsychotic that is one of the most potent drugs to treat schizophrenia (Asenjo Lobos et al., 2010). The stock solution of clozapine was prepared in 0.1 N HCl and diluted in phosphate-buffered saline. All mice groups were treated with 5 mg/kg clozapine (Sigma) intraperitoneally for 10 days. Control mice received only vehicle. After administration, the mice had a resting period followed by behavioral tests as described in Figure 2A.

### Gelatin Gel Zymography

Gel zymography with gelatin as a substrate was performed to detect and compare MMP-9 activity. Briefly, protein extracts that contained active MMPs were prepared from the whole brain (primarily the cortex and hippocampus) of *Mmp-9* heterozygotes and their wt counterparts and were analyzed by gelatin gel zymography using a previously described method (Lepeta et al., 2017). The gels were stained with Coomassie Blue. The intensity of lighter colored bands that corresponded to MMP-9 and MMP-2 was analyzed using ImageJ software. MMP-9 activity was determined after normalization to MMP-2 activity.

### Statistical Analyses

The behavioral data were analyzed using GraphPad Prism 6.0 software and two-way analysis of variance (ANOVA), unpaired *t*-test, and the nonparametric Mann-Whitney *U*-test. The data are expressed as mean ± standard error of mean (SEM) for samples with *n* > 5.

## Results

The present study investigated the effects of low MMP-9 activity on the predisposition to schizophrenia-like symptoms in mice that were subjected to psychosocial stress. We employed a milder modification of the resident intruder paradigm to induce psychosocial stress in mice, that appears to be an ethologically appropriate social defeat paradigm (Adamcio et al., 2009). *Mmp-9* heterozygous mice were exposed to the stress procedure; cognitive deficits and negative and positive schizophrenia symptoms were assessed (Figure 1A). We used only one test to assess positive symptoms (i.e., MK-801-induced hyperlocomotion), one test to assess cognitive deficits (i.e., novel object recognition test), and two tests to evaluate negative symptoms in *Mmp-9* heterozygous mice. We performed these four tests only to ensure that behavioral testing did not fatigue the mice.

The tail-suspension test was used to evaluate behavioral despair (i.e., one characteristic negative symptom of schizophrenia). Stressed *Mmp-9* heterozygotes were immobile for a significantly longer time (individual *t*-test, *p* < 0.05; two-way ANOVA, *F*_(1,37)_ = 7.008, *df* = 1, *p* < 0.05), compared with both stressed wt animals and non-stressed heterozygous littermates (Figure 1B), suggesting a diminished ability of stressed *Mmp-9* heterozygotes to cope with the adverse conditions. Similarly, in the social interaction test, stressed *Mmp-9* heterozygous mice spent a significantly shorter time interacting with a conspecific gender-matched mouse (individual *t*-test, *p* < 0.01; two-way ANOVA, *F*_(1,37)_ = 8.9555, *df* = 1, *p* < 0.05), compared with both stressed wt mice and non-stressed *Mmp-9* heterozygous mice (Figure 1C), indicating a reduction of sociability. These results indicate the development of negative-like symptoms of schizophrenia in *Mmp-9* heterozygous mice that were subjected to psychosocial stress.

To evaluate cognitive deficits, the mice underwent the novel object recognition test, which evaluates spatial learning and memory. *Mmp-9* heterozygous mice exhibited learning and memory impairments, reflected by a significant decrease in preference to explore the object in a novel location (individual *t*-test, *p* < 0.05), compared with the familiar location (Figure 1D). Interestingly, stress did not potentiate this performance impairment in *Mmp-9* heterozygotes.

MK-801 is an NMDA receptor antagonist that produces positive-like symptoms of schizophrenia in rodents (van den Buuse, 2010). MK-801-treated *Mmp-9* heterozygous mice exhibited hyper-locomotion (individual *t*-test, *p* < 0.05; two-way ANOVA, *F*_(1,37)_ = 14.61, *df* = 1, *p* < 0.005), compared with their wt littermates (Figure 1E). These findings are consistent with a previous report (Lepeta et al., 2017). Notably, stressed wt mice also exhibited an increase in locomotion, but stress exposure did not accentuate this effect in *Mmp-9* heterozygous mice.

Finally, we analyzed the enzymatic activity of MMP-9 by gel zymography in all four groups of mice before and after behavioral testing. As expected, *Mmp-9* heterozygous mice exhibited a two-fold decrease in MMP-9 activity. In stressed *Mmp-9* heterozygotes, MMP-9 activity decreased by more than 70% compared with stressed wt mice (Figure 1F; individual *U*-test, *p* < 0.05; two-way ANOVA, *F*_(1,8)_ = 13.9, *df* = 1, *p* < 0.05).

Typical and atypical antipsychotics have been traditionally used to treat schizophrenia (Asenjo Lobos et al., 2010). Thus, we investigated whether the negative-like schizophrenia symptoms that were observed in stressed *Mmp-9* heterozygous mice could be treated with an antipsychotic. Clozapine is an atypical antipsychotic that is used to treat patients with schizophrenia. In the present study, it was administered for 10 days after behavioral training (Figure 2A). The treatment abolished the increase in immobility that was observed in stressed heterozygous mice in the tail suspension test (Figure 2B; individual *t*-test *p* > 0.05; two-way ANOVA *F*_(1,18)_ = 0.1414, *df* = 1, *p* > 0.05). Vehicle treatment did not exert an effect in stressed heterozygous mice (Figure 2B; *t*-test, *p* < 0.05). Vehicle treatment also did not alter the reduction of social interaction in stressed *Mmp-9* heterozygous mice (Figure 2C; *t*-test, *p* < 0.05). However, clozapine ameliorated the effect of stress in *Mmp-9* heterozygotes, with no significant differences in interaction time between groups (Figure 2C; individual *t*-test *p* > 0.05; two-way ANOVA *F*_(1,18)_= 0.0132, *df* = 1, *p* > 0.05). Therefore, clozapine rescued negative-like symptoms of schizophrenia in our experimental model.

## Discussion

The present study found that psychosocial stress that was induced in the resident-intruder paradigm and low levels of MMP-9 were collectively required to evoke negative-like symptoms of schizophrenia in mice, indicated by: (i) an increase in immobility in the tail suspension test, reflecting by a decrease in the ability of stressed heterozygous mice to cope with the adverse conditions; and (ii) a shorter interaction time with a gender-matched conspecific mouse, reflecting social withdrawal. We found no influence of gene × environment interactions on cognitive deficits or positive-like symptoms of schizophrenia, which have been previously shown to be affected by lower MMP-9 activity alone (Vafadari et al., 2016). Importantly, studies of *MMP-9* genetic polymorphisms in human schizophrenia patients have shown that the gene variants predisposing to lesser MMP-9 expression confer higher disease risk (Rybakowski et al., 2009; Lepeta et al., 2017). The present study showed that stress exacerbated schizophrenia-like symptoms in mice that had low MMP-9 levels, thus supporting the hypothesis of the involvement of MMP-9 in schizophrenia and suggesting a possible pharmacological treatment target.

Schizophrenia can be categorized into deficit and non-deficit types, based on the occurrence of negative symptoms. Schizophrenia is considered a deficit type if it meets the criteria of more than two negative symptoms that are not fully accounted for by already existing depression and anxiety traits, effects of drugs, or environmental conditions (Kirkpatrick et al., 2017). The paradigm that we employed in the present study induced negative symptoms only in stressed *Mmp-9* heterozygous mice and not in wt mice, implying that, specifically, gene × environment interactions and not environmental influences alone are necessary for the observed phenotype. Thus, stressed *Mmp-9* heterozygous mice might serve as a useful model of the deficit type of schizophrenia. However, caution must be taken when considering the reliability of animal models of schizophrenia (Powell and Miyakawa, 2006; Nestler and Hyman, 2010; Young et al., 2010; Jones et al., 2011; Canetta and Kellendonk, 2018).

The positive symptoms of schizophrenia can be treated to varying degrees by antipsychotics, but negative and cognitive symptoms are often resistant to such treatment (Nestler and Hyman, 2010; Young et al., 2010; Jones et al., 2011). Some studies, however, have reported that some antipsychotics, such as clozapine, rescued negative symptoms to some extent (Brar et al., 1997; Remington et al., 2016; Khan and Zaidi, 2017). Such variable effects have been attributed to the fact that schizophrenia is a heterogeneous disorder, in which symptom severity and the treatment response vary greatly between patients. In the present study, we found that the endophenotypes exhibited by *Mmp-9* heterozygous mice that were exposed to psychosocial stress, particularly the negative symptoms, were rescued by clozapine. Thus, treatment with this drug could be considered in cases of schizophrenia patients who have specific *MMP-9* gene variations. The exact mechanism of action of clozapine remains to be elucidated- whether by targeting different pathways that are influenced by low MMP-9 levels, such as brain-derived neurotrophic factor (Huang, 2013) and glycogen synthase kinase 3β (Kang et al., 2004), or by directly affecting dendritic spine formation (Critchlow et al., 2006; Takaki et al., 2018).

The utility of mouse or other animal models of schizophrenia has been debated (Powell and Miyakawa, 2006; Nestler and Hyman, 2010; Young et al., 2010; Jones et al., 2011; Canetta and Kellendonk, 2018). Nonetheless, animal models that recapitulate environmental influences (e.g., stress, drugs, infections, and socioeconomic status) on clinically identified risk-factor genes in the development of schizophrenia symptoms, including cognitive, positive, and negative symptoms, are able to provide insights into possible gene × environment interactions. This is important for understanding the pathogenesis of schizophrenia and for identifying possible molecular targets for future research (Burrows and Hannan, 2016; Moran et al., 2016; Misiak et al., 2018).

Despite progress in understanding genetic and environmental risk factors for schizophrenia, little is known about the mechanisms of gene × environment interactions (Ayhan et al., 2016). The present study describes a novel mouse model of schizophrenia-related gene × environment interactions that involves the risk gene *Mmp-9*. Stress further decreased MMP-9 levels in heterozygous mice, which may be directly related to the endophenotype of negative symptoms of schizophrenia. However, further studies are required to understand whether such effects of environmental factors (i.e., stress) on *Mmp-9* gene expression occur through epistatic or epigenetic changes or broader signaling alterations. An interesting line of investigation would be to determine the ways in which low MMP-9 levels affect such behavioral changes, such as through aberrant synaptic plasticity (Michaluk et al., 2011; Vafadari et al., 2016) or neuroinflammation. Notably, MMP-9 has been reported to be involved in these phenomena (Müller et al., 2015; Vafadari et al., 2016; van Kesteren et al., 2017).

In conclusion, the present study validated the association between MMP-9 and schizophrenia symptoms. Low brain levels of MMP-9 appear to be a risk factor for schizophrenia, in which gene × environment interactions that involve *Mmp-9* contribute to the pathophysiology of this neuropsychiatric disorder.

## Data Availability

The datasets generated for this study are available on request to the corresponding author.

## Ethics Statement

### Animal Subjects

The animal study was reviewed and approved by Local Ethics Committee (Permission No. 148/2016 and 507/2018) Animal Protection Act in Poland, Directive 2010/63/EU.

## Author Contributions

BV, SM, and LK designed the study. BV and SM performed the experiments and analyzed the data. SM wrote the manuscript. MS and LK revised the manuscript. BV, SM, MS, and LK approved the final version of the manuscript.

## Conflict of Interest Statement

The authors declare that the research was conducted in the absence of any commercial or financial relationships that could be construed as a potential conflict of interest.
